# Suboptimal Blood Pressure Control, Associated Factors, and Choice of Antihypertensive Drugs among Type 2 Diabetic Patients at KCMC, Tanzania

**DOI:** 10.1155/2020/4376251

**Published:** 2020-07-20

**Authors:** Gabriel W. Mbwete, Kajiru G. Kilonzo, Elichilia R. Shao, Nyasatu G. Chamba

**Affiliations:** ^1^Department of Internal Medicine, Kilimanjaro Christian Medical University College, Moshi, Tanzania; ^2^Department of Internal Medicine, Kilimanjaro Christian Medical Centre, Moshi, Tanzania

## Abstract

**Background:**

Hypertension (HTN) can be present in up to two-thirds of patients living with diabetes mellitus (DM). It is a risk factor for the development of diabetes as well as complications like coronary artery disease (CAD), nephropathy, retinopathy, and neuropathy. Hypertension is treatable, and the degree to which it is controlled determines the risk of development of cardiovascular diseases and other complications in a given individual patient. Even though antihypertensive drugs are available and issued to hypertensive diabetic patients, the rate of control of HTN is often inadequate. The aim of this study was to assess the prevalence of suboptimal blood pressure (BP) control, its associated factors, and the choice of antihypertensive drugs among type 2 DM patients at Kilimanjaro Christian Medical Centre (KCMC).

**Methods:**

A hospital-based cross-sectional study was conducted at the KCMC diabetes clinic from October 2018 to March 2019 among type 2 DM patients with HTN based on the inclusion criteria. Data were collected using structured questionnaires, and written informed consent was obtained. Suboptimal BP was defined as BP levels ≥ 140/90 mmHg according to the American Diabetes Association guideline published in 2018. Data analysis was done using the Statistical Package for the Social Sciences (SPSS) version 25. Chi-square analysis was done to identify the independent predictors of BP control, and a *p* value of <0.05 was considered to be statistically significant.

**Results:**

The data of 161 participants was analysed; the mean age was 63.9 ± 20.2 years, with the majority being females (67.1%). Despite all participants being on different classes of antihypertensives, 57.8% had suboptimal BP control. Among the participants with good BP control, 52.7% were on angiotensin-converting enzyme inhibitors (ACE-I). Poor diabetes control was observed in 50.1% participants as indicated by elevated glycated haemoglobin.

**Conclusion:**

This study demonstrated that BP control in type 2 DM patients was suboptimal in more than half of the participants. The study showed that the use of ACE-I or angiotensin II receptor blockers (ARBs) in the majority of DM patients has a good impact in the control of blood pressure. The early initiation of ACE-I or ARBs among the diabetic patients will improve the optimal BP control.

## 1. Introduction

Diabetes mellitus (DM), more simply called diabetes, is a group of metabolic diseases characterised by hyperglycaemia resulting from defects in insulin secretion, insulin action, or both.

According to the International Diabetes Federation (IDF) report published in 2017, DM is estimated to affect over 425 million people or 8.8% of adults aged 20-79 years worldwide and expected to rise to 629 million by the year 2045. Africa is estimated to have 16 million people living with DM, which is predicted to increase to 41 million people by the year 2045. Approximately 87% to 91% of all people with DM have type 2 DM especially in the high-income countries. [1]

Hypertension (HTN) in patients living with DM is contributed by insulin resistance, obesity, and inactivity [2]. Studies have shown that 40% of individuals with type 2 DM already have HTN at the time of diagnosis and the remaining percentage develop hypertension later in life [3]. Both HTN and DM are noncommunicable diseases (NCDs) which are extremely common and frequently coexist. They are major contributors to the development and progression of macrovascular complications such as coronary artery diseases, stroke, and peripheral artery diseases and microvascular complications which include nephropathy, neuropathy, and retinopathy in people with DM compared to the general population [4].

The growing burden of DM and HTN globally is considered to be a major medical and public health problem associated with increased morbidity and mortality. Nonoptimal BP control is the leading cause of deaths globally responsible for 7 million deaths annually of which most are from low- and middle-income countries (LMIC) [5].

HTN is a major modifiable risk factor for cardiovascular diseases (CVDs) including heart failure and myocardial infarction, estimated to cause 65% deaths globally [6]. Lowering BP is particularly beneficial in diabetic patients; however, there is no specific level of lowering BP when cardiovascular and renal complications start to occur; thus, the definition of HTN is arbitrary and needed for practical reasons in patient assessment and treatment. The knowledge that reduction of BP in severe HTN reduces complications implies long-term supervision of therapy for many hypertensive patients so as to ensure adequate control of BP in order to prevent the complications of a poorly controlled BP [7]. There is no threshold for the risk of CVD, but rather a continuous decrease in risk as blood pressure is reduced.

The Appropriate BP Control in DM (ABCD) study, the United Kingdom Prospective Diabetes Study (UKPDS), and the Hypertension Optimal Treatment (HOT) trial showed a consistent benefit on cardiovascular events or mortality when BP is reduced. The number of patients with diabetes in Tanzania is increasing. Prevalence of HTN among patients with diabetes is equally high [8]. Furthermore, diabetes patients with poorly controlled HTN have a greater risk of delayed onset complications and poor prognosis of both conditions.

The degree of control of HTN in both rural and urban areas of Tanzania is not well established; therefore, effective BP control in DM patients is an important goal that demands special attention. The American Diabetes Association (ADA) guidelines published in 2018 recommends blood pressure targets of <140/90 mmHg in patients with diabetes [9]. This study was aimed at determining the prevalence of suboptimal BP control among diabetic patients and the associated risk factors as well as at evaluating appropriate antihypertensive medication to manage HTN in diabetic patients.

## 2. Methods

A hospital-based cross-sectional analytical study was conducted at the Kilimanjaro Christian Medical Centre (KCMC) diabetes clinic from October 2018 to March 2019 among type 2 DM patients with HTN. KCMC is a consultant referral hospital in north-eastern Tanzania. It caters to a wide population estimated to be over 15 million in the northern part of the country. The diabetes clinic is run once a week with an average of 100 patients on each clinic day.

Patients with type 2 DM above 18 years old with established clinical diagnosis of hypertension and on regular medications were considered eligible and enrolled consecutively until the desired sample size of 161 was attained. The diagnosis of HTN was defined as BP ≥ 140/90 mmHg based on the ADA guideline published in 2018 [9] and the JNC 7 guideline which categorised HTN as stage 1 BP 140/90-159/99 mmHg and stage 2 BP ≥ 160/100 mmHg; those participants who were on antihypertensive medications according to the patients' files were also considered hypertensive. Patients who were too sick to provide written informed consent and pregnant women were excluded from the study.

### 2.1. Data Collection

A face-to-face interview was conducted, after obtaining written informed consent. A structured questionnaire was used to collect the patients' sociodemographic characteristics and the duration of HTN and DM. The history of drug compliance and regular clinic attendance was obtained from the patients' files, and the attendance of three months consecutively was regarded as regular. Physical activity was assessed using the International Physical Activity Questionnaires (IPAQ). This is the validated tool for self-reported measure of physical activity. After 5 minutes of rest, the BP was measured using the Spengler sphygmomanometer; then, a second reading was taken after 5 minutes of the first reading, and the third BP reading was obtained from the patients' last medical visit records, and thus, the overall average BP was obtained. The drug medications were self-reported using the single item past 7 days by [10]. Suboptimal BP was defined as BP levels ≥ 140/90 mmHg according to the American Diabetes Association guideline published in 2018 for the management of hypertension in patients with DM [9]. The body mass index (BMI) for participants was calculated by obtaining the weight in kilograms (kg) and height in meter squared (m^2^). Random blood glucose (RBG) was measured using a standardized Gluco Plus machine (Glucometer Type 25 KB JPG) capillary finger prick method, and further venous blood samples were drawn for serum creatinine, serum urea and fasting total cholesterol, triglycerides, high-density lipoprotein cholesterol (HDL-C), and low-density lipoprotein cholesterol (LDL-C) and for glycated haemoglobin (HBA1C), and lastly midstream urine for protein was taken.

### 2.2. Data Management and Analysis

Data analysis was done using the Statistical Package for the Social Sciences (SPSS) version 25.

Categorical variables were expressed as proportions and percentages while continuous variables were expressed as means ± standard deviation. Comparison between categorical variables was done by the use of the chi-square test, while that of numerical variables was done by the use of analysis of variance; multivariates were used to assess the strength of association between the independent and dependent variables in the study, and a *p* value of <0.05 was considered to be statistically significant. We categorised BP as normal when it was <140/90 mmHg, stage 1 HTN when it was 140/90 mmHg to 159/99mmHg, and stage 2 HTN when it was ≥160/100 mmHg.

## 3. Results

The data of 161 participants were analysed in this study. The majority of the study participants were females 108 (67.1%), and the mean age of the participants was 63.9 ± 20.4 years; the majority of them 124 (77%) were aged between 50 and 75 years. The highest level of education attained by most of the participants was primary level which accounted for 59.6%, and 76 (47.2%) of the respondents were either peasants or small-scale farmers; the majority of them were either current drinkers or those who used to take alcohol, accounting for 67.8%, and cigarette smoking was observed in 14 (8.7%) of the respondents. Among the respondents, 67.7% had taken initiative measures of reducing salt intake in their meals. Two-thirds of the participants were covered by the National Health Insurance Fund (NHIF) in Tanzania. Other subscribers from different insurance companies were not considered because they were temporarily suspended by the hospital management during the time of the study (see Table 1).

In the medical and laboratory characteristics shown in Table 2, the family history of hypertension among the participants was 30.4% and 37.3% have been hypertensive for more than 5 years. Physical inactivity was reported in 6.2% of the participants, and 83 (51.6%) were overweight. The total serum cholesterol was normal in 63.2% with 51.5% of triglycerides. Urine for protein was negative in 80.8%, and normal serum creatinine was reported in 128 (79.5%) of the participants. 50.3% of the participants had uncontrolled blood sugar for the past three months prior to this study as demonstrated by the HBA1C as shown in Table 2.

### 3.1. Social Characteristics in Association with Suboptimal Blood Pressure Control

The prevalence of suboptimal BP in this study was 57.8%. A high prevalence was obtained from male patients accounting for 60.4% and in patients aged 75 years and above (68%) (*p* ≤ 0.482), and retired participants had 70.8% (*p* ≤ 0.079) suboptimal BP control. Cigarette smoking was another factor which was associated with suboptimal BP control (as shown in Table 3).

**Figure 1:**
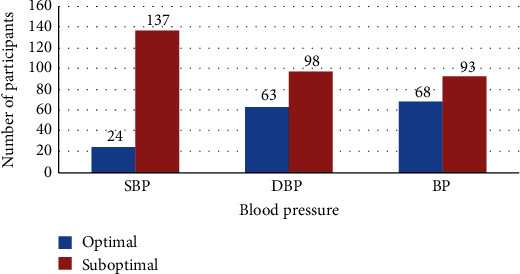
Overall blood pressure control among the participants.

The overall suboptimal BP control according to the systolic BP (SBP) and diastolic BP (DBP) was seen in 137 (85.1%) and in 98 (60.9%), respectively. Thus, the overall suboptimal SBP/DBP was 93 (57.8%) as shown in Figure 1.

### 3.2. Clinical and Laboratory Characteristics of the Participants in Association with Suboptimal Blood Pressure Control

A family history of HTN of the respondents was significantly associated with suboptimal blood pressure control (71.4%) (*p* ≤ 0.020). Duration since the diagnosis of HTN or DM was another factor which contributed to suboptimal BP control as those who had HTN for 11 years and those who had above 5 years since DM diagnosis were 65.8% and 60%, respectively. Overweight and obesity were also associated with suboptimal BP control (56.6% and 63.2%, respectively). Suboptimal BP control was reported in 90.0% of the participants who were physically inactive, and in those who were having few days of physical activities (1-5 days per week), suboptimal BP control accounted for 59.1%. The total serum cholesterol was normal in 63.2%. Positive urine for protein was found in 21% with uncontrolled BP. Elevated serum creatinine was reported in 45.5% of the participants with suboptimal BP control whereas uncontrolled blood sugar as indicated by high HbA1C levels was recorded in 64.9% (*p* ≤ 0.078) of the participants. Drug adherence was another predictor of suboptimal BP control as was reported in 81.8% (*p* ≤ 0.004) of the participants (see Table 4).

All the participants were on hypertensive treatment therapy. Renin-angiotensin system blocking drugs, i.e., angiotensin-converting enzyme inhibitors (ACE-I) or angiotensin II receptor blockers (ARBs) or ACE-I/ARBs in combination with other drugs, were the most frequently prescribed antihypertensive drugs; 72.1% of the participants were on those regimens.

Significant BP control was seen in participants who were receiving ACE-I alone (63.2%); even in the multivariate logistic regression, there were no changes. The poor control of HTN was observed in patients who were on calcium channel blockers alone (88.2%) (*p* ≤ 0.001).

**Figure 2:**
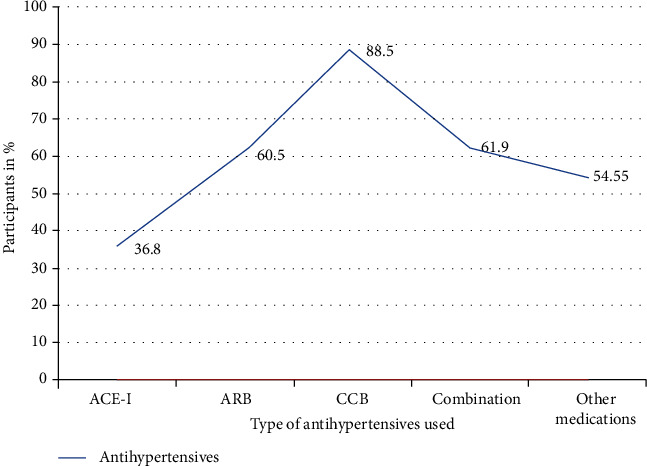
The antihypertensives used in association with suboptimal blood pressure control among the participants.

There was no big difference in suboptimal blood pressure control between patients who were taking oral hypoglycaemic drugs and those who used insulin (68.6% and 67.3%, respectively) (see Figure 2).

The following independent variables age, sex, level of education, salt reduction, family history of HTN, and type of antihypertensive drugs showing a significant association with the dependent variable in the univariate analysis therefore were used in the multivariate analysis as shown in Table 5.

## 4. Discussion

In this hospital-based cross-sectional analytical study, we explored the extent of suboptimal BP control as defined by the ADA 2018 guideline to be BP ≥ 140/90 mmHg [9].

The overall suboptimal BP control was 57.8%, whereby the prevalence of suboptimal systolic and diastolic BP control was 85% and 60.9%, respectively. This high prevalence is contributed by poor drug adherence and financial constraints for those paying out of their pockets, taking into consideration that in Tanzania, we have less than 15% of insured population. Suboptimal BP control has been reported in other many studies worldwide; to mention few, in India, uncontrolled BP was found to be 62.3% [11]; in the study done in Malaysia, suboptimal BP control was 51.75% [12]; and in the USA, 51.2% of the participants were found to have uncontrolled BP [13]. This is probably because we used the same cross-sectional methods.

The highest prevalence of suboptimal BP was observed in a study done in Singapore with 86.6% [14]. This is because it was a community-based study; i.e., there is a difference in the study population. The lowest findings were in the Netherlands with prevalence of 15% [15] and in the study done in Romania with prevalence of 10% [16]; this is because of the different methodology used in our study, they used cohort, and the retrospective nature of the study, respectively.

Suboptimal BP control was more prevalent in the elderly (56.5% and 68% among patients aged 50-75 years and above 75 years, respectively). These findings have been similar to those of other studies done in South Africa [17] and Ethiopia [18] which indicated an association of poor BP among the elderly. The explanation for this may be due to the increase in arterial stiffness and poor adherence to medication associated with advancing age, so the recommended cutoff point of BP in DM patients of <140/90 mmHg is too tight to the elderly people.

An increased body mass index as indicated by overweight and obesity was another important finding which was associated with suboptimal BP control where the prevalence was 56.6% and 63.2%, respectively. This was similar to the findings in Jordan where the prevalence of overweight and obesity was 52.5% and 28.8%, respectively [19].

Duration since the diagnosis of HTN in this study was also associated with suboptimal BP control, where 65.8% of the participants with the duration of more than 11 years were compared to those who had less duration. This was also observed in the study done in Ethiopia where the prevalence was 46.6% for participants who had duration of more than 5 years [18]. The difference is because a wide range of age categories was used. Likewise, a family history of hypertension was shown to have a strong association with suboptimal BP control by having 71.4% prevalence (*p* ≤ 0.02), it was different from the study done in Ethiopia which found the prevalence of 17.56% [18].

Uncontrolled blood sugar as shown by HBA1C was 68.2% which can be explained by poor adherence to medication. Elevated cholesterol was found in 50.3%; these findings are almost the same with that of the study done in Jordan (HBA1C (52.5%) and cholesterol (54.9%)) [19]. Inactivity and consumption of saturated fats in meat and cooking oils are the major factors contributing to dyslipidaemia.

Alcohol intake was another factor which contributed to suboptimal BP control among the study participants (56.9%); this is because most of the participants in our study were drinking local brew together with medication thinking that local brew was not alcohol. 87% was found in the study done in South Africa [17].

ACE inhibitors were found to be superior (53.57%) in the control of BP in diabetic patients to other antihypertensives; this has been observed in many studies including the study done in Germany which showed superiority of RAS blockade, particularly ACE inhibitors [20] and the study done in Oman that found that patients who were using ACE inhibitors (60% and 85%, respectively) had controlled pressure [21]. The use of calcium channel blockers alone was significantly associated with suboptimal BP control (88.2%). The combination of two or more drugs did not achieve treatment targets, but we still emphasize the use of more than one drug as it has been shown to have significance in various studies.

## 5. Conclusion

In this study, only 42.2% of hypertensive diabetic patients met the recommended BP control values in DM (<140/90 mmHg). More effort should be addressed to control the risk factors associated with poor BP control in diabetic patients. Physicians should put more emphasis on the early initiation and proper selection of antihypertensive especially the use of ACE inhibitors because they have shown better effect. It should be borne in mind that ACE-I and ARBs should not be taken concurrently.

### 5.1. Study Limitations

Most of the participants who attend the clinic every 2 to 3 months tend to refill their antihypertensive in other peripheral health facilities where there are limited drug options so compliance to one class or a course of antihypertensive cannot be guaranteed.

It was difficult to ascertain the contribution of dietary salt intake in the study population because we did not measure urine sodium excretion. Poor adherence to medication contributed by high cost of people who are not covered by health insurance was difficult to control.

## Figures and Tables

**Table 1:** Sociodemographic characteristics of the participants (*n* = 161).

Variables	*N*	%
Sex		
Male	53	32.9
Female	108	67.1
Age		
<50	12	7.5
50-75	124	77
75+	25	15.5
Level of education		
None	3	1.9
Primary	96	59.6
Secondary+	62	38.5
Occupation		
Employed	37	23
Peasant/farmer	76	47.2
Retired	48	29.8
Salt reduction in food		
No	52	32.3
Yes	109	67.7
Smoker		
No	147	91.3
Yes	14	8.7
Drink alcohol		
No	52	32.3
Yes	109	67.7
Who pays for the medication		
NHIF	142	88.2
Cash	19	11.8

**Table 2:** Clinical and laboratory characteristics of the participants (*n* = 161).

Variables	*N*	%
Duration since hypertension diagnosis		
<5 years	63	39.1
5-10 years	60	37.3
11+ years	38	21.6
Hypertension family history		
No	112	69.6
Yes	49	30.4
Duration since diabetes diagnosis		
<5 years	29	18
5-10 years	70	43.5
11+ years	62	38.5
Diabetes family history		
No	53	32.9
Yes	108	67.1
Physical activity		
None	10	6.2
1-5 days	88	54.7
>5 days	63	39.1
BMI		
Normal	40	24.8
Overweight	83	51.6
Obese	38	23.6
Low-density lipoprotein		
Normal	103	64
High	58	36
Triglycerides		
Normal	83	51.5
High	78	48.5
Total cholesterol levels		
Normal	102	63.2
High	59	36.8
HBA1C		
Normal	80	49.7
High	81	50.3
Serum creatinine		
≤106 *μ*mol/L	128	79.5
>106 *μ*mol/L	33	20.5

**Table 3:** Participants' sociodemographic characteristics in association with suboptimal blood pressure control (*n* = 161).

Variables	Optimal BP		Suboptimal BP		Total	Chi-square
	*N*	%	*N*	%		*p* value
Age						
<50	6	50	6	50	12	
50-75	54	43.5	70	56.5	124	1.4577
75+	8	32	17	68	25	0.482
Sex						
Male	21	39.6	32	60.4	53	0.2212
Female	47	43.5	61	56.5	108	0.638
Level of education						
None	1	33.3	2	66.7	3	
Primary	37	38.5	59	61.5	96	1.5960
Secondary+	30	48.4	32	51.6	62	0.450
Occupation						
Employed	19	51.4	18	48.6	37	
Peasant/farmer	35	46.1	41	53.9	76	5.0744
Retired	14	29.2	34	70.8	48	0.079
Salt reduction in food						
No	17	32.7	35	67.3	52	2.8675
Yes	51	46.8	58	53.2	109	0.090
Smoker						
No	63	42.9	84	57.1	147	0.2673
Yes	5	35.7	9	64.3	14	0.605
Drink alcohol						
No	15	39.5	23	60.5	38	0.1556
Yes	53	43.1	70	56.9	123	0.693

**Table 4:** Clinical and laboratory characteristics in relation to suboptimal blood pressure (*n* = 161).

Variables	Optimal BP		Suboptimal BP		Total	Chi-square
	*N*	%	*N*	%		*p* value
HTN family history						
No	54	48.2	58	51.8	112	5.3909
Yes	14	28.6	35	71.4	49	0.020
Duration of hypertension						
<5 years	28	44.4	35	55.6	63	
5-10 years	27	45	33	45	60	1.3170
11+ years	13	34.2	25	65.8	38	0.518
Duration of diabetes						
<5 years	13	44.8	16	55.2	29	
5-10 years	28	40	42	60	70	0.2671
11+ years	27	43.5	35	56.5	62	0.875
Physical activity						
None	1	10	9	90.0	10	
1-5 days	36	40.9	52	59.1	88	5.5775
>5 days	31	49.2	32	50.8	63	0.061
Body mass index						
Normal	18	45	22	55	40	
Overweight	36	43.4	47	56.6	83	0.6224
Obese	14	36.8	24	63.2	38	0.733
Low-density lipoprotein						
Normal	45	43.7	58	56.3	103	0.2475
High	23	39.7	35	60.3	58	0.619
Triglycerides						
Normal	39	47	44	53.0	83	1.5857
High	29	37.2	49	62.8	78	0.208
Total cholesterol levels						
Normal	46	44.2	58	55.8	104	0.4791
High	22	38.6	35	61.4	57	0.489
HBA1C						
Normal	41	48.8	43	51.2	84	3.1108
High	27	35.1	50	64.9	77	0.078
Drug adherence						
Never missed	37	61.7	23	38.3	60	
Missed ≤3 days	35	38.9	55	61.1	90	11.189
Missed >3 days	2	18.2	9	81.8	11	0.004
Serum creatinine						
≤106 *μ*mol/L	50	39.06	78	60.9	128	2.5779
>106 *μ*mol/L	18	54.6	15	45.5	33	0.108

**Table 5:** Determinants of suboptimal blood pressure in multivariate analysis (*n* = 161).

Variables	Crude estimates		Adjusted estimates	
	OR (95% CI)	*p* values	AOR (95% CI)	*p* values
Sex				
Male	Ref		Ref	
Female	0.85 (0.44, 1.66)	0.638	1.07 (0.5, 2.33)	0.858
Age				
<50	Ref		Ref	
50-75	1.3 (0.4, 4.24)	0.668	1.5 (0.36, 6.04)	0.588
75+	2.1 (0.52, 8.7)	0.295	2.53 (0.46, 13.94)	0.286
Level of education				
None	Ref		Ref	
Primary	0.8 (0.07, 9.1)	0.855		
Secondary+	0.53 (.05, 6.19)	0.615		0.450
Salt reduction in food				
No	Ref		Ref	
Yes	0.55 (0.28, 1.1)	0.092	0.55 (0.24, 1.22)	0.142
Antihypertensive				
ACE inhibitors	Ref		Ref	
ARB	2.63 (1.13, 6.11)	0.025	2.27 (0.94, 5.46)	0.068
CCB	12.86 (3.98, 41.59)	<0.001	10.9 (3.29, 36.09)	<0.001
Combination therapy	2.79 (0.99, 7.82)	0.052	2.27 (0.77, 6.72)	0.139
Other medications	2.1 (0.56, 7.57)	0.278	1.64 (0.42, 6.38)	0.475
Family history of HTN				
No	Ref		Ref	
Yes	2.33 (1.13, 4.79)	0.022	1.83 (0.82, 4.1)	0.142

## Data Availability

The database used during this study analysis will be available on request from the corresponding author.

## Abbreviations

ABCD: Appropriate Blood Pressure Control in Diabetes
ACE-I: Angiotensin-converting enzyme inhibitors
ADA: American Diabetes Association
ARB: Angiotensin II receptor blockers
BMI: Body mass index
CVDs: Cardiovascular diseases
DBP: Diastolic blood pressure
DM: Diabetes mellitus
HBA1C: Glycosylated haemoglobin
HDL-C: High-density lipoprotein cholesterol
HOT: Hypertension Optimal Treatment
HTN: Hypertension
IDF: International Diabetes Federation
JNC 7: Joint National Committee, seventh report
KCMC: Kilimanjaro Christian Medical Centre
LDL-C: Low-density lipoprotein cholesterol
NCDs: Noncommunicable diseases
NHIF: National Health Insurance Fund
SBP: Systolic blood pressure
UKPDS: United Kingdom Prospective Diabetes Study
WHO: World Health Organization.
